# Neurons in the pigeon nidopallium caudolaterale, but not the corticoidea dorsolateralis, display value and effort discounting activity

**DOI:** 10.1038/s41598-019-52216-3

**Published:** 2019-10-30

**Authors:** Madeline Dykes, Blake Porter, Michael Colombo

**Affiliations:** Department of Psychology, University of Otago, Dunedin, New Zealand

**Keywords:** Motivation, Reward

## Abstract

We recorded from single neurons in two areas of the pigeon brain while birds were required to peck a stimulus indicating either a high effort task or a low effort task would follow. Upon completion of the task the birds received the same reward. We found that activity in the nidopallium caudolaterale, an area equivalent to the mammalian prefrontal cortex, was modulated by the value of the reward that would be received based on how much effort was required to obtain it. Value coding was most prominent during the presentation of the stimulus indicating a high or low effort task, and in the delay period immediately prior to carrying out the effort task. In contrast, activity in the corticoidea dorsolateralis was not modulated by value, however, population firing patterns suggest that it may be involved in associating actions with outcomes. Our findings support the view that activity in the nidopallium caudolaterale reflects value of reward as a function of effort discounting and as such may serve functions similar to the mammalian anterior cingulate cortex.

## Introduction

The value of an outcome is not based purely on the amount of reward expected, but also the costs that are required to obtain it. A number of studies have addressed the effects of effort cost in decision making paradigms by manipulating the effort required to achieve a reward. For example, rats placed in a T-shaped maze having to decide between receiving a large reward associated with a high-effort cost (climbing a barrier; HCHR) or a small reward associated with a low-effort cost (no barrier; LCLR) chose the HCHR arm on the majority of trials^1^. The authors also found that when the barrier was increased in size, the preference shifted to favour the LCLR arm, indicating a change in the perception of the reward value as effort requirements were manipulated^1^. Similar changes in behaviour have also been shown in primates^2^. Overall, these data support the theory that the value of an outcome is discounted based on the effort cost required to obtain that outcome.

A number of studies in rats and humans indicate the anterior cingulate cortex (ACC), a region of the prefrontal cortex, is critical to processing effort-based information^3–5^. The ACC is an area connected to a number of sites in the brain including the ventral midbrain, amygdala, and motor areas, and is thought to play a vital role in learning and updating information to guide decision making^6,7^. These features may indicate that the ACC is involved in decisions regarding the cost of physical effort. Hillman and Bilkey^8^, and Cowen, Davis, and Nitz^9^ recorded from neurons in the ACC of freely moving rats trained to choose between HCHR and LCLR arms in a maze, where climbing a barrier represented the effort cost. Hillman and Bilkey^8^ found that 63% of cells in the ACC increased firing prior to moving towards a specific reward outcome, and 94% of these cells fired more before moving towards the “best” option – in the first instance, HCHR rather than LCLR arm. When they manipulated the amount of reward given and the presence of barriers in both arms, ACC cells dynamically adjusted their firing rate to represent the most valuable option^8^. Cowen *et al*.^9^ reported similar patterns of activity, with ACC cells firing in relation to the most valuable option. These electrophysiological findings provide a neurological basis for effort-based decision making in the ACC of rodents, similar to that seen in humans.

A more recent study by Porter, Hillman, and Bilkey^10^ recorded from ACC cells in rats during a task where the effort requirements were manipulated but the reward amounts remained constant. The design differed from previous studies of effort discounting because it did not require the rats to make a choice between high and low effort. The rats ran along a shuttle box that was tilted at different angles, creating different levels of effort required to get to the reward. Recordings from the ACC again demonstrated the same dynamic response patterns seen in their earlier study in that ACC firing was responsive to the most valuable actions and outcomes accounting for different effort states, but this time in a non-choice paradigm. The authors also found that cells fired differentially between high effort and low effort trials not only when they were carrying out the action, but also when they were receiving the reward^10^. These data indicate that the ACC may have a broader role in monitoring the effort costs of behaviours and their outcomes regardless of decision making demands.

A number of behavioural studies in birds have shown that manipulating effort costs also change the perceived value of the associated reward^11,12^, suggesting that the same neural mechanisms may underlie effort-based decisions in the avian brain. Studies in chicks focussing on the neural basis of effort have found that lesions to the arcopallium and the medial striatum decrease both foraging efforts and choices to exert greater effort to retrieve food, even when the food amount is large^13–16^. However, the neural encoding of effort discounting has not yet been explored in the pigeon brain. Given that in mammals the frontal areas are crucial to value discounting by effort costs, we examined whether the nidopallium caudolaterale (NCL), an area in the avian brain that has been defined as the equivalent of the mammalian prefrontal cortex (PFC)^17,18^, and whose neurons have been shown to code reward amount and reward value^19–22^, would serve a similar function in birds.

Pigeons were trained on high-effort (HE) and low-effort (LE) tasks. The HE task required the birds to peck a total of eight times across four spatially-distributed positions on the screen, whereas the LE task required four pecks to a single central location on the screen. If NCL neurons predicted the most valuable outcome, as they do in the mammalian ACC, then we would expect to see more firing towards a stimulus that predicts an upcoming LE task than HE task. We balanced the stimuli used for each condition across the birds so that any elevation in firing rate was due to the effort manipulation rather than simply visual selectivity. In addition to recording from NCL we also recorded from the area corticoidea dorsolateralis (CDL). CDL is a thin area on the outermost layer of the brain connecting the caudal pallium and the hippocampus^23^. Atoji and Wild^23^ (see also Csillag & Montagnese^24^) have compared the CDL to the cingulate cortex of the mammalian brain. For example, CDL has connections to the hippocampal complex, basal ganglia, and amygdala, as does the cingulate cortex in mammals. Unlike the mammalian cingulate cortex, however, CDL does not connect to higher order motor areas and to the brainstem, and CDL has connections to olfactory areas that do not exist in the cingulate cortex^23^. To our knowledge, electrophysiological recordings have not been carried out in CDL. The effort task used in the current experiment provided an opportunity to explore any functional similarities between CDL and the mammalian cingulate cortex in encoding the value of effortful behaviours.

## Methods

### Subjects

The subjects were six experimentally naïve adult homing pigeons (*Columba livia*). The birds had free access to grit and water and were maintained at 80–85% of their free feeding body weight and fed a mixture of wheat, corn, peas, pellets, and grains. They were housed in individual wire mesh cages with a 12 h to 12 h light–dark cycle beginning at 07:00 h. The subjects were kept and treated in accordance with the University of Otago Code of Ethical Conduct for the Manipulation of Animals and the University of Otago Animal Ethics Committee approved the experiment.

### Apparatus

The apparatus and the methods used were similar to that used by Dykes *et al*.^25^. The pigeons were trained and tested in standard operant chambers measuring 35 cm (length) x 43 cm (width) x 39 cm (height). At the front of the chamber was a 17-inch monitor. In front of each monitor sat a Carroll Touch infrared touch frame (EloTouch, baud rate 9600, transmission time 20 ms). In front of the touch frame sat a plexiglass panel with six square holes arranged in a 2-row by 3-column format. The size of each square hole was 6 cm × 6 cm and the center-to-center distance of each hole was 6.5 cm. Situated 20 cm below the center key was a hopper that could be illuminated and delivered the wheat reward. The stimuli used were four black and white pictures; a picture of a cactus flower, a picture of Arnold Schwarzenegger, a picture of a person on a skateboard, and a picture of a black crow. Each bird was assigned one of the pictures to represent high effort (HE) trials and a different picture to represent low effort (LE) trials. The stimuli used to predict HE and LE trials were balanced across the birds. The stimuli were 6 cm × 6 cm in size and appeared centered in the square hole. Every peck to the touch screen was accompanied by a 100 ms, 1000-Hz tone.

### Behavioural task

**Figure 1:**
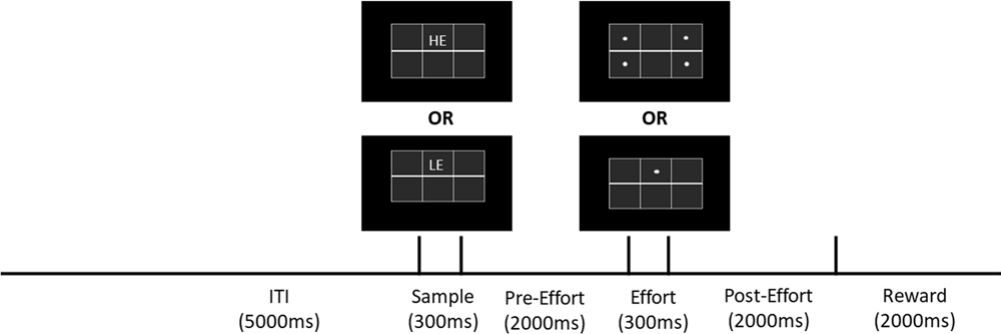
Behavioural procedure. At the end of the ITI, either a high-effort (HE) or low-effort (LE) sample stimulus was presented in the centre top hole. Three pecks to the stimulus turned it off and initiated a 2 sec Pre-Effort period. On HE trials, four dots appeared, one in each corner of the screen. The bird was required to peck each dot twice before being rewarded. On LE trials, the bird was simply required to peck one dot in the middle of the screen four times. Following the Effort period was a 2 sec Post-Effort period, followed by 2 sec of access to reward.

At the end of a 5 sec intertrial interval (ITI), one stimulus appeared in the top center square hole (see Fig. 1). Three pecks to the stimulus turned it off and initiated a 2 sec Pre-Effort delay period, followed by either a high-effort (HE) or low-effort (LE) Effort period. On HE trials four dots appeared, one in each of the top-left, top-right, bottom-left, and bottom-right holes, and the pigeon was required to peck each dot twice. The pigeon was allowed to peck the dots in any order, and each dot disappeared after it had been pecked twice. On LE trials, a dot appeared in the top center hole and the pigeon was required to peck it four times. If the pigeon pecked at a dot location after the dot had disappeared, the peck tone sounded, but the trial sequence was not otherwise interrupted. After all dots had been pecked in the Effort period there followed a 2 sec Post-Effort period, followed by a Reward period during which the pigeon was given 2 sec access to wheat, irrespective of whether the effort condition was HE or LE. Each session consisted of 64 trials with 32 trials dedicated to each of the stimuli, randomly intermixed.

### Surgery

After behavioural training the birds were implanted with a lightweight microdrive to allow single-unit recording. They were anesthetized with a mixture of ketamine (25 mg/kg) and xylazine (5 mg/kg). After pruning the feathers on the scalp and overlaying the ears, the head was immobilized using a Revzin stereotaxic adapter^26^. The scalp was first sprayed with a topical anesthetic (10% xylocaine) and the skin overlying the skull was cut and retracted to expose the skull. For the four NCL birds, a hole was drilled above the NCL at AP +5.5, and ML ±7.5^27^. The tips of the electrodes of the microdrive were lowered to position them above the NCL. For CDL birds, the microdrive was built so that the electrodes were angled at 27° and the electrodes lowered into the brain so the tips were at AP +6.0 and ML ±4.0. Six stainless steel screws were placed into the skull (one serving as an electrical ground screw). The microdrive was attached to the skull with dental acrylic. The incision was sutured and sprayed with xylocaine. The bird recovered in a padded and heated cage until fully alert and mobile before returned to its home cage. There it was allowed to recover for at least seven days before the recording sessions started.

### Neural recording

Eight 25 µm formvar-coated nichrome wires mounted in the microdrive were used for recording the extracellular activity of single neurons in the NCL. For each session one electrode was used to record the neural activity and a second electrode with minimal activity served as the indifferent. The signal was passed through a FET headstage, then a Grass P511K preamplifier (Grass Instruments, Quincy, Massachusetts, United States) where it was amplified and filtered to remove 50 Hz noise. An oscilloscope and speaker were used to monitor the signals. A CED micro1401 system (Cambridge Electronic Design Limited, Cambridge, United Kingdom) collected the electrophysiological data, and CED Spike2 software was used for behavioural time-tagging of all events and analysis of the spike data. Good isolation of a cell and a signal-to-noise ratio of at least 2:1 were the criteria for cell selections.

After cell isolation the behavioural program was started. A recording session lasted approximately 45 minutes. At the end of the session, the electrodes were advanced approximately 40 µm. The birds were tested once a day.

### Data analysis

Data analysis was similar to that used in Dykes *et al*.^25^ The 300 ms sample period (Sample), the 2 sec Pre-Effort period (Pre-Effort), the 300 ms effort period (Effort), the 2 sec Post-Effort period (Post-Effort) and the 2 sec reward period (Reward) were all subject to analysis. With respect to the Sample period, unlike studies with primates where one can monitor the exact position of the eyes, it is difficult in pigeons to monitor when they are looking at the visual stimulus. Colombo, Frost, and Steedman^28^ adopted a convention that neural activity to a visual stimulus was measured during a period from −400 ms to −100 ms prior to the first peck to that stimulus. The reason the period ends 100 ms prior to contact with the keys is because pigeons close their eyes approximately 80 ms prior to a key peck^28^. In the ITI, the Pre-Effort, and the Post-Effort periods, the first 500 ms of each period was excluded from analysis in order to avoid including residual activity driven by the period that took place immediately before (Reward, Sample, and Effort periods, respectively). With respect to the Effort period analysis, the LE and HE periods differed in both latency to complete the effort requirement and in the number of pecks required. Therefore, the 300 ms period prior to making the first effort peck was analysed rather than then entire Effort period.

All cells that fired at less than 0.1 spikes/sec in the ITI were excluded from further analysis. Each cell’s Sample, Pre-Effort, Effort, Post-Effort, and Reward period data was subjected to a two-way repeated-measures ANOVA with period (2: ITI and defined period) and stimulus (2: HE and LE) as factors. The dependent variable was the firing rate on each trial of a cell during the ITI and defined period. An effect of Stimulus indicated that the cell responded differently on HE and LE trials. A Period effect indicated that a cell either increased or decreased its firing rate in the defined period compared to the ITI. The cell’s data was entered into a population plot on the basis of whether the main effect of Stimulus (HE vs LE) was significant, thereby illustrating the firing pattern of cells that differentiate between HE and LE trials in a given period.

Those cells that did show an effect of stimulus were defined as “Effort Selective”. In other words, they fired differentially to LE and HE trials. We then also examined whether the Effort Selective cells, as a group, showed a LE Value Preference. In order to establish whether they showed a LE Value Preference, the data in the Sample, Pre-Effort, Effort, Post-Effort, and Reward period of the sub-population of all Effort Selective cells for the defined period was subject to a repeated-measures two-way ANOVA with Stimulus (2: HE or LE) and Bin (all bins 50 ms, 6: bins 1–6 for the Sample and Effort periods; 30: bins 1–30 for Pre-Effort and Post-Effort periods; 40: bins 1–40 for the Reward period) as factors, with repeated measures over bins (Greenhouse-Geisser corrected). If the sub-population of Effort Selective cells fired significantly more during LE trials compared to HE in the defined period, they were characterized as having a LE Value Preference.

## Results

### Histology

**Figure 2:**
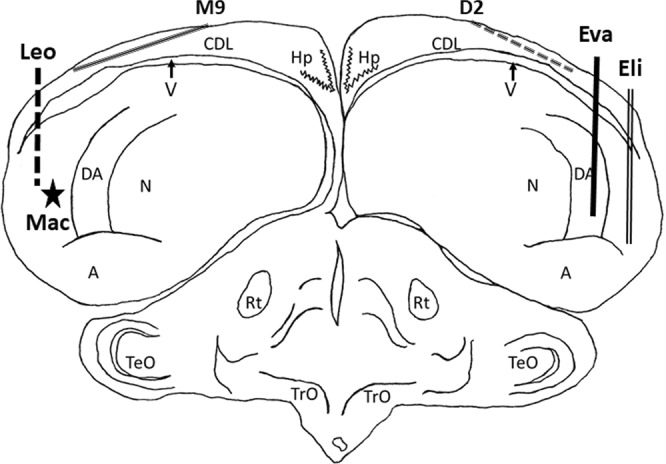
Electrode track reconstruction. Electrode track position reconstructions for the two right NCL birds (Eli and Eva), the right CDL bird (D2), one left NCL bird (Leo), and one left CDL bird (M9). All recordings were within the full dorsal-ventral extent of NCL and CDL. We were unable to recover the electrode tracks of the second left NCL bird (Mac), but the termination point indicated by the depth records is represented by the star. The following are the brain regions as defined by Reiner *et al*.^34^ A, arcopallium; DA, tractus dorso-arcopallialis; CDL, area corticoidea dorsolateralis; Hp, hippocampus; N, nidopallium; Rt, nucleus rotundus; TeO, tectum opticum; TrO, tractus opticus; V, ventricle.

For NCL and CDL birds all electrode tracks were within the targeted region as defined by Karten and Hodos^26^, and the histology results are shown in Fig. 2. The intended track positions for NCL electrodes were AP +5.5 and ML ±7.5. The track position for the right hemisphere NCL bird Eli was AP +6.75 and ML +8.9, differing from the intended AP position by 1.25 mm and the intended ML position by 1.4 mm. The track position for the right hemisphere NCL bird Eva was AP +6.25 and ML +8, differing from the intended AP position by 0.75 mm and the intended ML position by 0.5 mm. The track position for the left hemisphere NCL bird Leo was AP +6 and ML −9, differing from the intended AP position by 0.5 mm and the intended ML position by 1.5 mm. We were unable to identify the electrode tracks in the remaining left hemisphere bird, Mac, although the entry point was located at AP +5.3, differing from the intended AP position by only 0.2 mm. The intended entry point for CDL electrodes were AP +6 and ML ±4. The entry point for the right hemisphere CDL bird D2 was AP +6.75 and ML +4, differing from the intended AP position by 0.75 mm. The entry point for the left hemisphere CDL bird M9 was AP +6.5 and ML −3.5, differing from the intended AP position and ML position by 0.5 mm.

### Behavioural data

**Figure 3:**
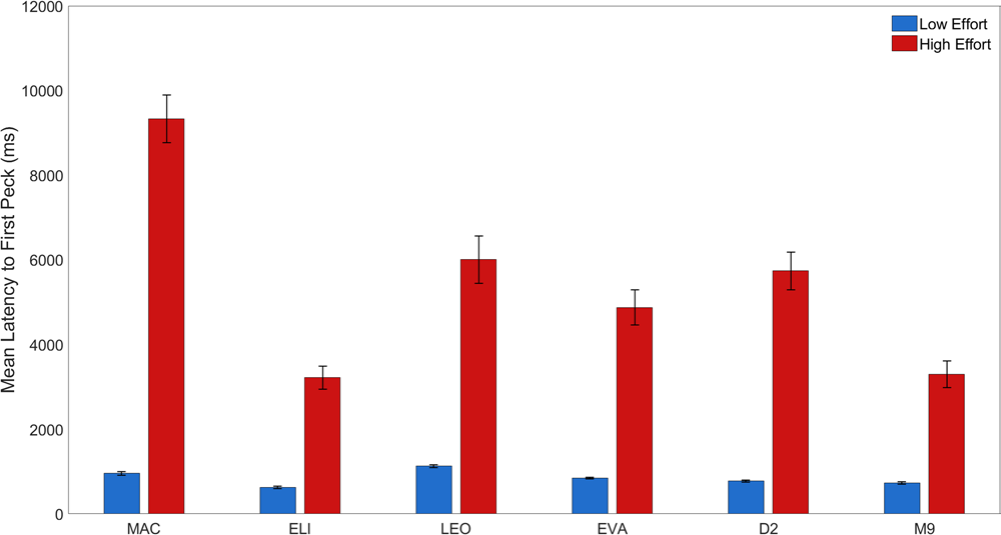
Behavioural performance. Mean latency to the first peck for the LE and HE stimuli for each of the four NCL birds (Mac, Eli, Leo, and Eva) and two CDL birds (D2 and M9), averaged across sessions during which neurons were recorded. Note that the shorter latencies indicate higher value to the pigeon. Error bars represent ± 1 SEM.

For each session, the median latency to the first peck of the sample stimulus (indicating either high or low effort) was calculated across the 32 trials dedicated to each stimuli. The latency to the first peck averaged across all sessions from which cells were recorded is displayed in Fig. 3. The latencies for each bird were subjected to a Wilcoxon’s signed rank test and in all cases, the latency to the stimulus indicating a HE trial was significantly longer than the latency to the LE stimulus (Mac: *t*(39) = 15.40, *p* < 0.001; Leo: t(43) = 8.92, *p* < 0.001; Eva *t*(31) = 9.98, *p* < 0.001; Eli t(44) = 9.85, *p* < 0.001; D2: t(29) = 11.01, *p* < 0.001; M9: t(20) = 7.64, *p* < 0.001).

### Period selective and effort selective cells

We recorded from a total of 245 NCL cells. Four cells were removed on the basis that their average firing rate across the entire ITI period was less than 0.1 spikes per second, leaving 241 cells that were used for the NCL analysis. We recorded from a total of 57 cells in CDL. Two cells were removed because the firing rate in the ITI was less than 0.1 spikes per second, leaving 55 cells that were used for the CDL analysis. A repeated-measures two-way ANOVA with Stimulus (2: HE or LE) and Period (2: the defined period and ITI) as factors was carried out on each of the NCL and CDL cells, separately. The results are shown in Table 1.

**Table 1:** Period Selective and Effort Selective cells in each trial period.

Trial Period	NCL	NCL	CDL	CDL
	Period Selective	Effort Selective	Period selective	Effort Selective
Stimulus	67 (28%)	31 (13%)	16 (29%)	3 (6%)
Pre-Effort (peck adjusted)	100 (42%)	34 (14%)	23 (42%)	8 (140.5%)
Effort	135 (59%)	23 (10%)	52 (95%)	2 (4%)
Post-Effort	185 (77%)	26 (11%)	47 (86%)	8 (15%)
Reward	174 (72%)	25 (10%)	40 (73%)	10 (18%)

### Effort selective cells in the Sample period

#### NCL cells

**Figure 4:**
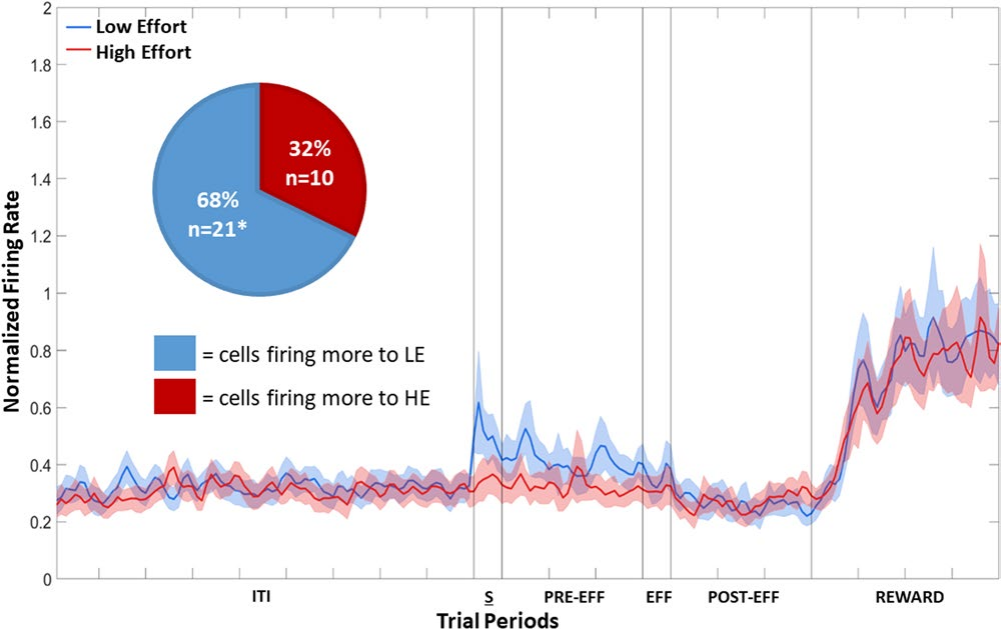
Population plot: Effort Selective NCL cells in the Sample period. Normalised firing rate for Effort Selective cells during the Sample period. The ITI represents the last 4500 ms of the 5000 ms intertrial interval. The Sample period (S) represents the 300 ms period prior to the first peck to the stimulus. The Pre-Effort period (PRE-EFF) represents the 1500 ms before the effort stimuli appear, and the Effort period (EFF) represents the 300 ms prior to the first effort peck. The Post-Effort period (POST-EFF) is the 1500 ms prior to reward delivery, and Reward represents the 2000 ms reward delivery period. ITI: intertrial interval; S: Stimulus period; PRE-EFF: Pre-Effort period; EFF: Effort period; POST-EFF: Post-Effort period.

**Figure 5:**
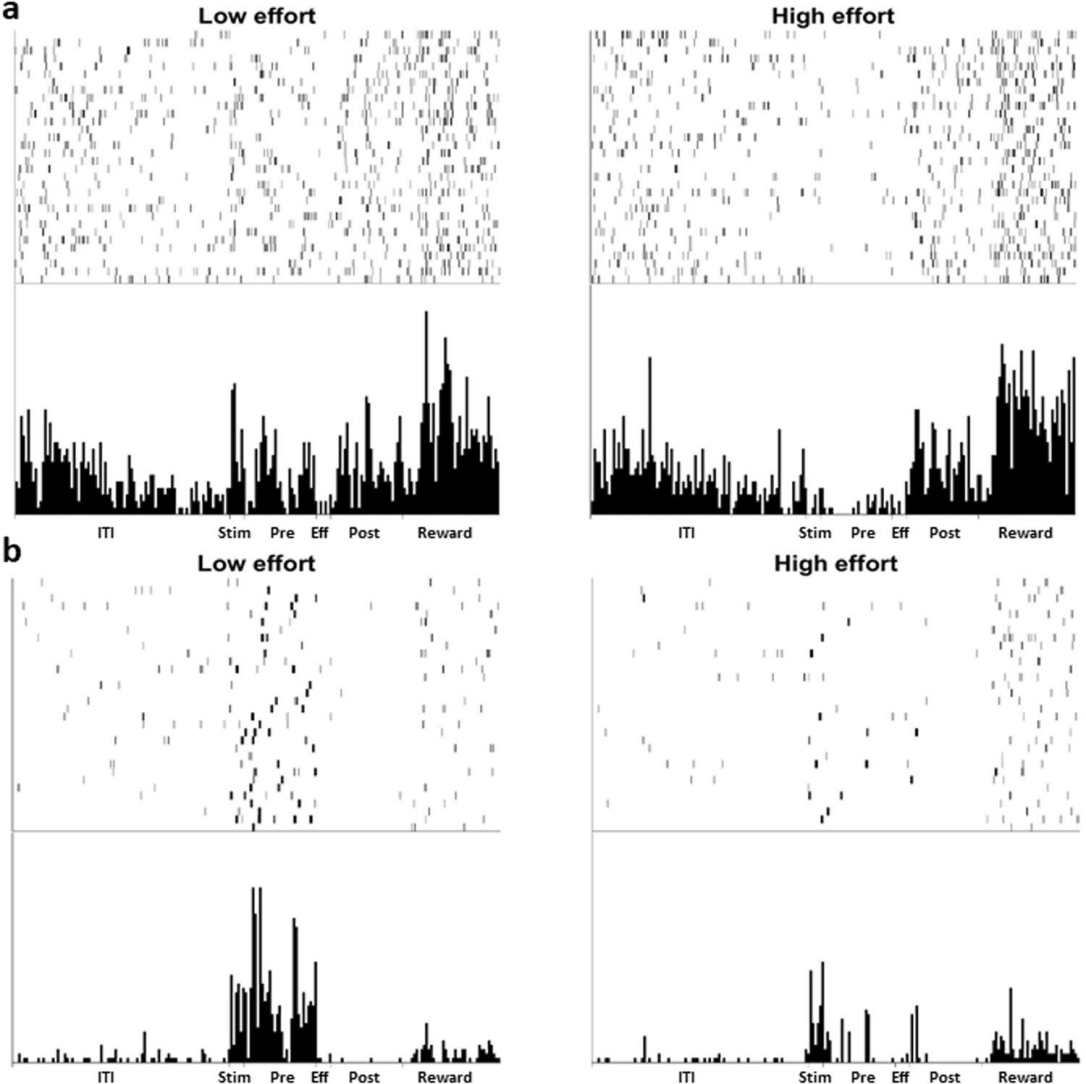
Examples of NCL single-unit activity. Panel (a,b) show two different cells, each from different birds. Each panel displays the raster (top) and histogram (bottom) activity of the cell over a 64 trial session. (**a**) An NCL cell that displays a noticeable increase in firing to the presentation of the LE stimulus compared to the HE stimulus. The cell also fires in an inhibitory manner during the Pre-Effort period of HE trials. (**b**) An NCL cell that displays an increased firing rate during the Pre-Effort period of LE trials, but not during HE trials. ITI: intertrial interval; Stim: Stimulus period; Pre: Pre-Effort period; Eff: Effort period; Post: Post-Effort period.

The population plot of the 31 Effort Selective NCL cells, irrespective of whether they fired more in HE or LE trials, is shown in Fig. 4. Twenty one of the cells fired more to the LE stimulus, while the remaining 10 cells fired more in response to the HE stimulus. A Chi-squared test revealed that the number firing more to LE than HE was greater than expected by chance, *X*^2^ (1, n = 31) = 3.90, *p* < 0.05. The data in the Sample, Pre-Effort, Effort, Post-Effort, and Reward period was subject to a repeated-measures two-way ANOVA, with Stimulus (2: HE or LE) and Bin (all bins 50 ms, 6: bins 1–6 for the Sample and Effort periods; 30: bins 1–30 for Pre-Effort and Post-Effort periods; 40: bins 1–40 for the Reward period) as factors, with repeated measures over bins (Greenhouse-Geisser corrected). There was a significant effect of Stimulus during the Sample period, *F*(1, 30) = 4.49, *p* < 0.05, and in the Pre-Effort period, *F*(1, 30) = 6.46, *p* < 0.05, with cells firing more during LE trials than HE trials, and therefore the Effort Selective cells, as a population, showed a LE Value Preference. There was no effect of Stimulus, in the Effort, Post-Effort, and Reward period, all *F*s(1, 30) < 0.8, all *p*s > 0.38. An example of an Effort Selective NCL cell in the Sample period is shown in Fig. 5a. In CDL, only three cells were Effort Selective and so given the small number, no further analysis was conducted.

### Effort selective cells in the Pre-Effort period

#### NCL cells

**Figure 6:**
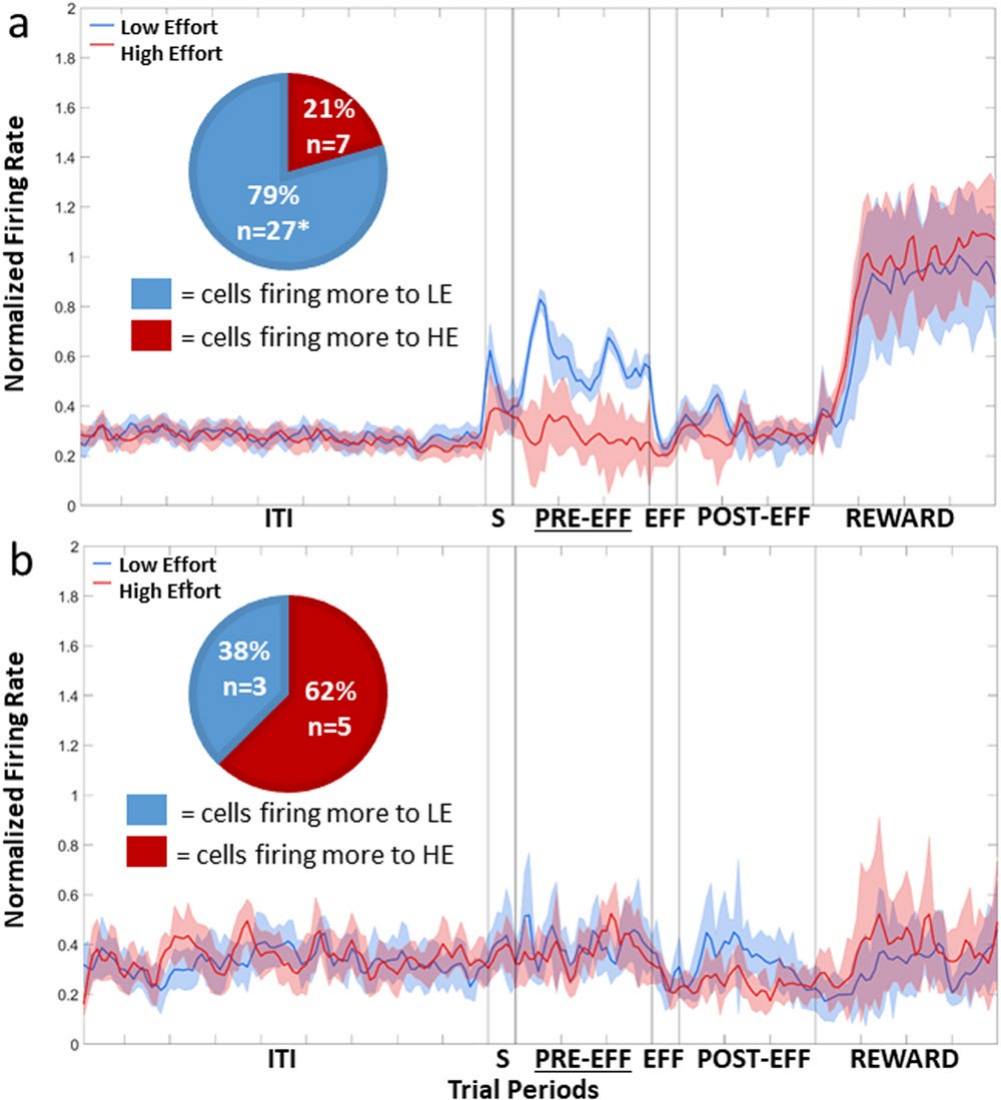
Population plot: Effort Selective Cells in the Pre-Effort period. (**a**) Normalised firing rate for Effort Selective NCL cells during the Pre-Effort period, correcting for peck related activity. (**b**) Normalised firing rate for the Effort Selective CDL cells during the Pre-Effort period, correcting for peck related activity. For details on the timing of the periods, see Fig. 4. ITI: intertrial interval; S: Stimulus period; PRE-EFF: Pre-Effort period; EFF: Effort period; POST-EFF: Post-Effort period.

The activity of the 34 Effort Selective cells in the Pre-Effort period, after adjusting to control for peck related activity, irrespective of whether they fired more during HE or LE trials, is displayed in Fig. 6a. Twenty-seven of the 34 Effort Selective NCL cells fired more during LE trials, while seven fired more during HE trials. A Chi-squared test revealed that there were more cells that fired to LE than would be expected by chance, *X*^2^ (1, n = 34) = 11.77, *p* < 0.001. An example of an Effort Selective NCL cell in the Pre period is shown in Fig. 5b. The data in the Sample, Pre-Effort, Effort, Post-Effort, and Reward period was subject to a repeated-measures two-way ANOVA, with Stimulus (2: HE or LE) and Bin (all bins 50 ms 6: bins 1–6 for the Sample and Effort periods; 30: bins 1–30 for Pre-Effort and Post-Effort periods; 40: bins 1–40 for the Reward period) as factors, with repeated measures over bins (Greenhouse-Geisser corrected). There was a significant effect of Stimulus in the Sample period, *F*(1,33) = 4.80, *p* < 0.05, and the Pre-Effort period, *F*(1, 33) = 16.71, *p* < 0.001, with cells firing significantly more during LE trials than HE trials, and therefore the Effort Selective cells, as a population, showed a LE Value Preference. In the Effort period, there was a slight difference between firing in HE and LE trials, with more firing during LE trials, however the difference fell short of significance, *F*(1, 33) = 3.71, *p* = 0.06. There was no significant difference firing between HE and LE trials during the Post-Effort, and Reward periods, all *F*s(1, 33) < 2.16, all *p*s > 0.15.

#### CDL cells

The population plot of the eight Effort Selective CDL cells, after controlling for the possible effect of pecks, is shown in Fig. 6b. Of the eight cells, five fired more during HE trials, while the remaining three fired more during LE trials. A Chi-squared comparison revealed that no more cells fired to LE compared to HE than would be expected by chance, *X*^2^ (1, n = 8) = 0.5, *p* = 0.48. The data in the Sample, Pre-Effort, Effort, Post-Effort, and Reward period was subject to a two-way ANOVA, with Stimulus (2: HE or LE) and Bin (all bins 50 ms 6: bins 1–6 for the Sample and Effort periods; 30: bins 1–30 for Pre-Effort and Post-Effort periods; 40: bins 1–40 for the Reward period) as factors, with repeated-measures over Bins (Greenhouse-Geisser corrected). There was no significant effect of Stimulus in any period, all *F*s(1, 6) < 2.33, all *p*s > 0.17.

### Effort selective cells in the Effort period

#### NCL cells

**Figure 7:**
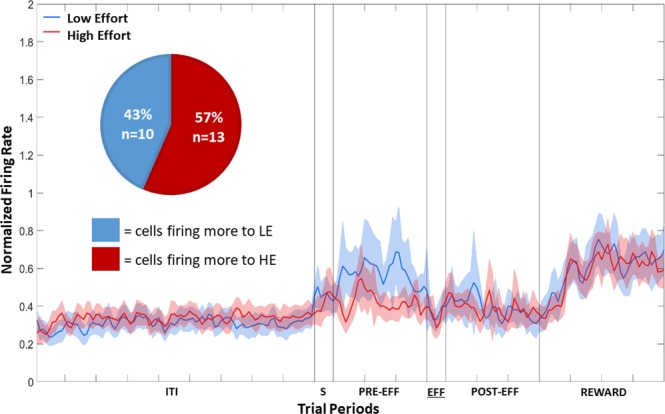
Population plot: Effort Selective NCL Cells in the Effort period. Normalised firing rate for Effort Selective NCL cells during the Effort period. For details on the timing of the periods, see Fig. 4. ITI: intertrial interval; S: Stimulus period; PRE-EFF: Pre-Effort period; EFF: Effort period; POST-EFF: Post-Effort period.

The population response of the 23 Effort Selective NCL cells, irrespective of whether firing was higher during LE and HE trials, is shown in Fig. 7. Ten cells fired more during LE trials, while the remaining 13 fired more in HE trials. A Chi-squared test revealed that there were no more cells firing more to HE to LE than would be expected by chance, *X*^2^ (1, n = 23) = 0.39, *p* = 0.53. The data in the Sample, Pre-Effort, Effort, Post-Effort, and Reward period was subject to a repeated-measures two-way ANOVA, with Stimulus (2: HE or LE) and Bin (all bins 50 ms 6: bins 1–6 for the Sample and Effort periods; 30: bins 1–30 for Pre-Effort and Post-Effort periods; 40: bins 1–40 for the Reward period) as factors, with repeated measures over Bins (Greenhouse-Geisser corrected). In the Sample, Pre-Effort, Effort, Post-Effort, and Reward periods, there was no effect of Stimulus, all Fs(1, 22) < 3.53, all *p*s > 0.07. In CDL, only two cells were Effort Selective and so given the small number, no further analysis was conducted.

### Effort selective cells in the Post-Effort period

#### NCL cells

**Figure 8:**
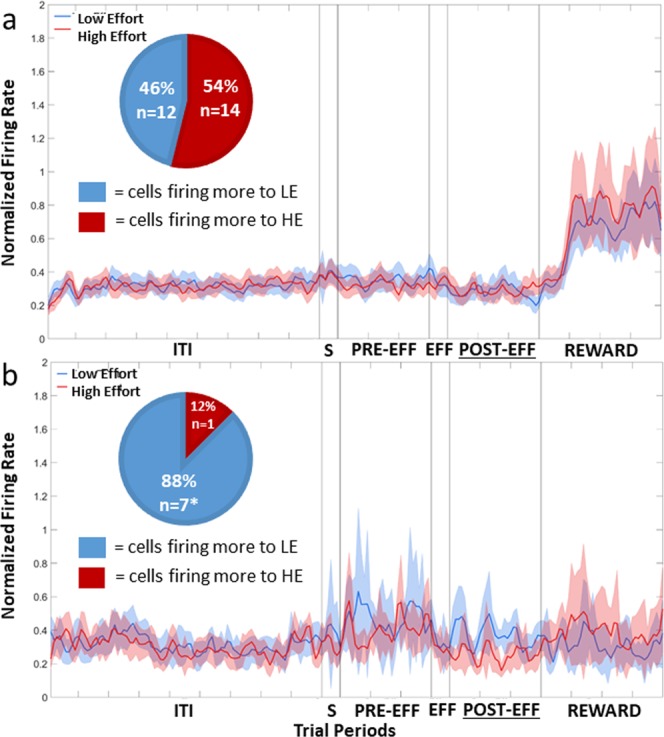
Population plot: Effort Selective Cells in the Post-Effort period. (**a**) Normalised firing rate for Effort Selective NCL cells during the Post-Effort period. (**b**) Normalised firing rate for Effort Selective CDL cells during the Post-Effort period. For details on the timing of the periods, see Fig. 4. ITI: intertrial interval; S: Stimulus period; PRE-EFF: Pre-Effort period; EFF: Effort period; POST-EFF: Post-Effort period.

The population response of all 26 Effort Selective NCL cells, regardless of whether they fired more during LE or HE trials is shown in Fig. 8a. Twelve fired more during LE trials, while the remaining 14 fired more in HE trials. A Chi-squared test revealed that there were no more cells firing more to HE compared to LE than would be expected by chance, *X*^2^ (1, n = 26) = 0.15, *p* = 0.70. The data in the Sample, Pre-Effort, Effort, Post-Effort, and Reward period was subject to a repeated-measures two-way ANOVA, with Stimulus (2: HE or LE) and Bin (all bins 50 ms 6: bins 1–6 for the Sample and Effort periods; 30: bins 1–30 for Pre-Effort and Post-Effort periods; 40: bins 1–40 for the Reward period) as factors, with repeated measures over Bins (Greenhouse-Geisser corrected). In the Sample, Pre-Effort, Effort, Post-Effort, and Reward periods, there was no effect of Stimulus, *F*s(1, 25) < 1.71, all *p*s > 0.29.

#### CDL cells

**Figure 9:**
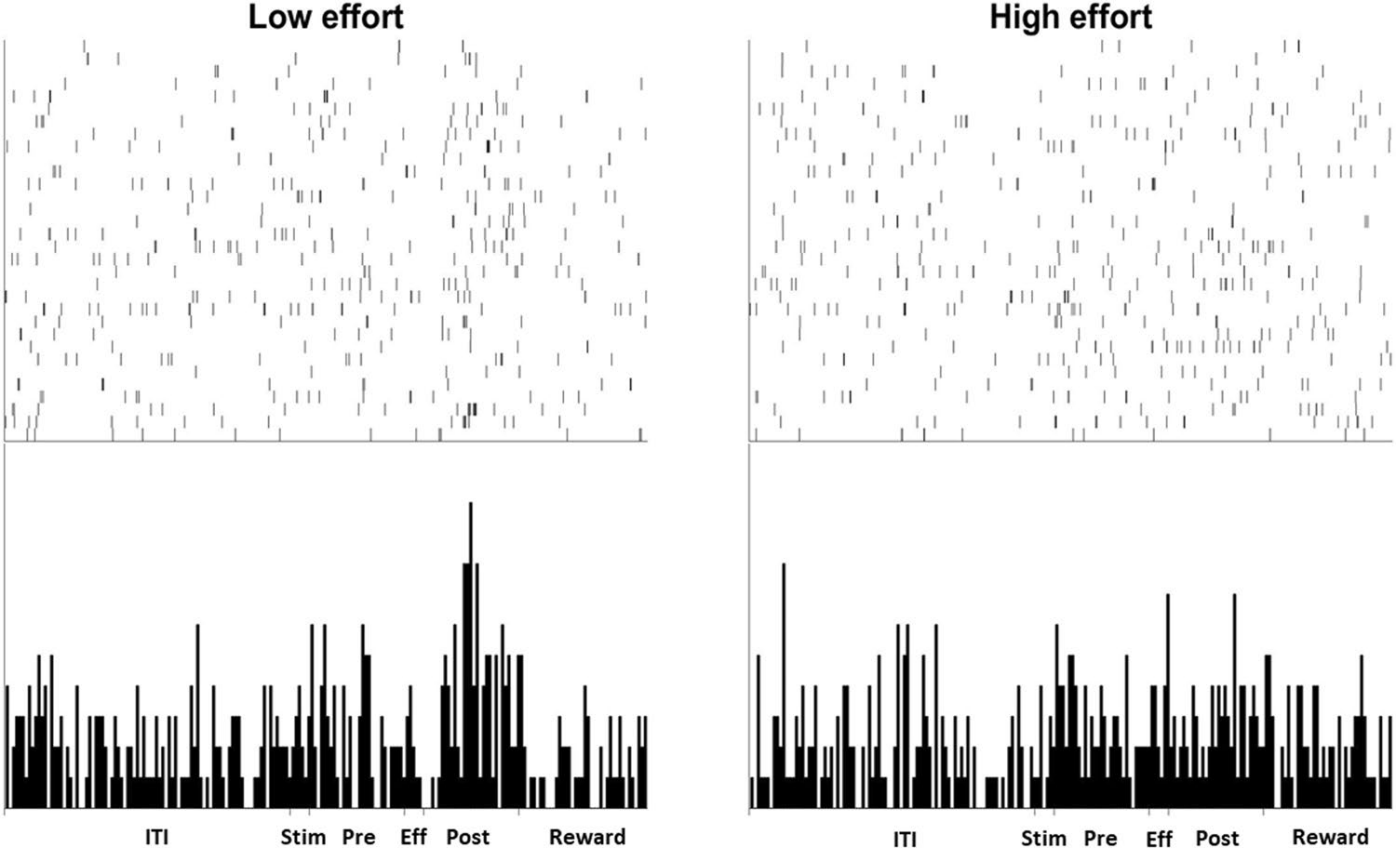
Examples of CDL single-unit activity. Each panel displays the raster (top) and histogram (bottom) activity of the cell over a 64-trial session. The cell displays a significant increase in firing during the Post-Effort period of LE trials compared to HE trials. ITI: intertrial interval; Stim: Stimulus period; Pre: Pre-Effort period; Eff: Effort period; Post: Post-Effort period.

The population plot of all eight Effort Selective CDL cells, irrespective of whether they fired more during HE or LE trials is shown in Fig. 8b. Of the eight Effort Selective cells, seven fired more during the Post-Effort period in LE trials, while the remaining cell fired more during the Post-Effort period in HE trials. An example CDL cell that shows a difference in firing between HE and LE trials in the Post-Effort period is shown in Fig. 9. A Chi-squared comparison revealed that more cells fired at higher rates during LE trials compared to HE trials than would be expected by chance, *X*^2^ (1, n = 8) = 4.5, *p* < 0.05. The data in the Sample, Pre-Effort, Effort, Post-Effort, and Reward period was subject to a repeated-measures two-way ANOVA, with Stimulus (2: HE or LE) and Bin (all bins 50 ms 6: bins 1–6 for the Sample and Effort periods; 30: bins 1–30 for Pre-Effort and Post-Effort periods; 40: bins 1–40 for the Reward period) as factors, with repeated measures over Bins (Greenhouse-Geisser corrected). There was no significant effect of Stimulus in any period, all *F*s(1, 7) < 1.67, all *p*s > 0.24.

### Effort Selective cells in the Reward period

#### NCL cells

**Figure 10:**
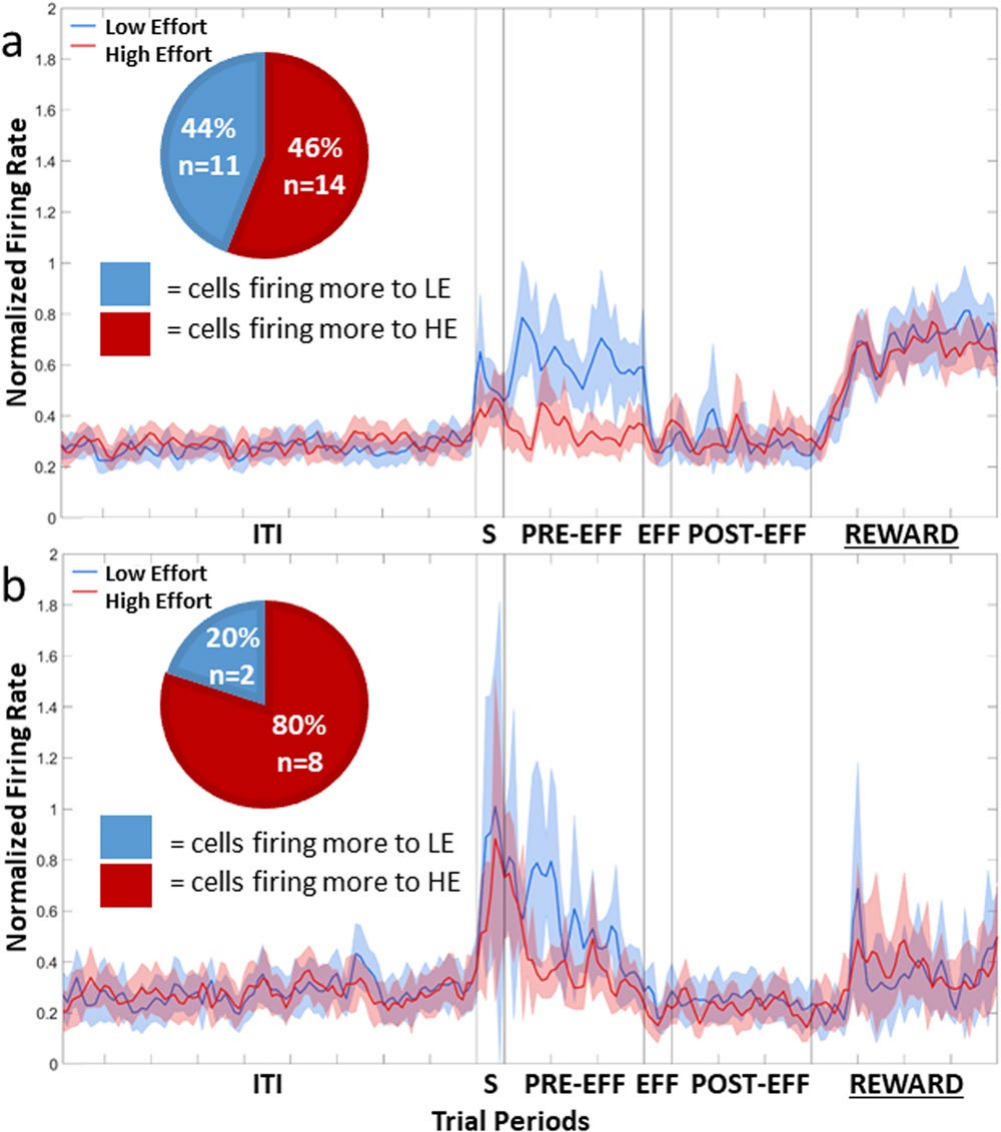
Population plot: Effort Selective Cells in the Reward period. (**a**) Normalised firing rate for Effort Selective NCL cells during the Reward period. (**b**) Normalised firing rate Effort Selective CDL cells during the Reward period. For details on the timing of the periods, see Fig. 4. ITI: intertrial interval; S: Stimulus period; PRE-EFF: Pre-Effort period; EFF: Effort period; POST-EFF: Post-Effort period.

The population response of all 25 Effort Selective NCL cells, irrespective of whether they fired more during HE or LE trials, is shown in Fig. 10a. Fourteen fired more during HE trials and 11 fired more during LE trials, and a Chi-squared test revealed that this was no more than would be expected by chance, *X*^2^ (1, n = 25) = 0.36, *p* = 0.55. The data in the Sample, Pre-Effort, Effort, Post-Effort, and Reward period was subject to a repeated-measures two-way ANOVA, with Stimulus (2: HE or LE) and Bin (all bins 50 ms 6: bins 1–6 for the Sample and Effort period, 30: bins 1–30 for Pre-Effort and Post-Effort periods, or 40: bins 1–40 for the Reward period) as factors, with repeated measures over Bins (Greenhouse-Geisser corrected). There was a significant effect of Stimulus in the Pre-Effort period, with cells firing more during LE than HE trials, *F*(1, 24) = 7.69, *p* < 0.05. In the Sample period, there was a slight difference between firing in HE and LE trial, with more firing during HE stimuli, however the difference fell short of significance, *F*(1, 24) = 4.11, *p* = 0.05. There was no significant effect of Stimulus in the Effort, Post-Effort, or Reward periods, all *F*s(1, 24) < 4.11, all *p*s > 0.05.

#### CDL cells

The population plot of all 10 Effort Selective cells, irrespective of whether they fired more during HE or LE trials, is shown in Fig. 10b. Of the 10 cells, eight fired more during the Reward period of HE trials, while the remaining two fired more during the Reward period in LE trials. A Chi-squared comparison revealed that the number of cells with higher firing rates during HE trials compared to LE just fell short of significance, *X*^2^ (1, n = 10) = 3.6, *p* = 0.06. The data in the Sample, Pre-Effort, Effort, Post-Effort, and Reward period was subject to a repeated-measures two-way ANOVA, with Stimulus (2: HE or LE) and Bin (all bins 50 ms 6: bins 1–6 for the Sample and Effort periods; 30: bins 1–30 for Pre-Effort and Post-Effort periods; 40: bins 1–40 for the Reward period) as factors, with repeated measures over Bins (Greenhouse-Geisser corrected). There was no significant effect of Stimulus in any period, all *F*s(1, 9) < 1.52, all *p*s > 0.25.

## Discussion

### Summary of findings

In the present study, we explored how NCL and CDL cells encode the value of a reward when it is discounted by effort costs. We recorded from 245 cells in NCL and 57 in CDL during a task where birds were required to peck a stimulus predicting a subsequent high effort task (peck a total of eight times across four spatially-distributed positions on the screen) or a subsequent low effort task (make four pecks to a single central location on the screen). The latency-to-peck data strongly supports the fact that the birds preferred the low effort trials over the high effort trials. We examined whether NCL and CDL cells exhibited Effort Selectivity, and, if so, whether or not they were Value Selective (fired preferentially to the Low Effort trials) in the period that the birds were presented with each stimulus that would predict the upcoming effort condition, the periods before and after exerting the effort, and the reward period.

Mirroring the behavioural preferences of the birds for the stimuli that predicted the LE trials, cells in NCL that were Effort Selective in both the Stimulus and the Pre-Effort periods fired significantly more during LE trials than HE trials, even when controlling for pecking. The stimuli indicating the trial types were balanced across birds, and we saw higher firing rates to the LE stimulus across all birds, therefore it is unlikely that the difference in firing rates was simply due to stimulus selectivity. In contrast to the Sample and Pre-Effort periods, Effort Selective cells showed no neural preference for LE trials over HE trials during the Effort, Post-Effort, or Reward periods. In contrast to NCL, while a number of CDL cells were Effort Selective, the population of Effort Selective CDL cells showed no increase in firing towards LE trials compared to HE trials during any period of the task, indicating no LE Value Preference in CDL cells.

### Implications for NCL function

Given that for both HE and LE trials the reward amount was the same but the physical effort different, the pattern of firing we saw in NCL is consistent with the notion that NCL is important for representing the effort-discounted value of a stimulus. Naturally, the LE and HE trials differ along a few other dimensions in addition to effort, and it is important to consider whether these factors could also be driving the observed neural differences. Given that NCL activity is modulated by the animals’ pecking^20^, a main factor to consider is whether higher peck rates to the LE stimulus that continued into the Pre-Effort period could account for the higher neural activity during the Sample and Pre-Effort periods. However, we do not believe this is likely for two reasons. First, in the Pre-Effort period, we statistically controlled for any peck-related neural activity, and even when doing so significant differences in neural activity emerged during the LE and HE trials. Second, the differences in neural activity were also present during the Sample period where, because the period of analysis was from −400 ms to −100 ms prior to the first peck, pecks had no bearing. Thus, the observed differences in NCL activity between HE and LE trials was likely driven by effort discounting of reward value and not an artefact of peck frequency.

The HE and LE trials also differ in terms of the spatial arrangements of the stimuli in that following the LE stimulus, the animal needs only to peck to one spatial position in the Effort period, whereas following the HE stimulus the animal needs to peck at four different spatial positions in the Effort period. Thus either the spatial positions themselves, or the movement differences that the two different spatial arrays would engender, might also account for the observed neural differences. Again, the same logic that we applied to our peck data would apply to an explanation based on differences in spatial arrays or movement. Although both differences in spatial arrays or movements could account for the differences in neural activity between LE and HE trials during the Pre-Effort period, neither could account for the differences in neural activity during the Sample period where the stimuli that predicted the LE and HE trials were presented in the same central position. Overall, we believe it was the impending difference in effort requirements that was driving the neural differences observed during the Sample and Pre-Effort periods.

A final point to consider is whether the longer latency to peck the HE stimulus compared to the LE stimulus could explain the difference in firing during the Sample period. The longer latency to peck the HE stimulus is a useful indicator of preference, or lack thereof, and our method of analysing the time period from −400 ms to −100 ms prior to the first peck is important because it is the only time we know the bird is looking at the stimulus. One possibility is that the longer latency to peck the HE stimulus could have resulted in neural habituation of the response to that stimulus. However, although neural habituation might explain the difference observed between LE and HE during the Sample period, it does not explain why we still see the difference during the Pre-Effort period. We are therefore confident that the nerual differences are meaningful and driven by the effort differences associated with the stimuli.

The apparent coding of value in NCL as a function of effort cost is similar to that seen in the ACC of mammals. In effort studies with rats, ACC cells fire to the “best” outcome when effort based options are manipulated^8,10,29^. While our task did not require dynamic changes in value appraisal, the current design allows us to see the encoding of a stimulus that, through conditioning, has been associated as the ‘better value’ option. Indeed, our findings in NCL are similar to Porter *et al*.^10^ who showed rodent ACC neural populations respond to behaviours with the highest value, even in effort tasks where no decision between behaviours needs to be made.

The literature supporting NCL as the functional analogue of the mammalian PFC is small, but growing. In respect to value coding, studies have found NCL to modulate firing in the same manner as the mammalian PFC in the anticipation of reward and reward delivery, as well as in response to the value of temporally discounted rewards^19,21,25^. To date, no one has explored whether value as a function of effort discounting is also encoded in NCL. The present study adds to the current knowledge in that it shows that NCL has properties similar to the mammalian ACC with respect to encoding the more valuable of two options when differing effort costs are required. Our findings are somewhat in contrast to where these functions are located in the mammalian frontal regions; unlike in the mammalian PFC, the same area of the avian NCL that encodes effort discounting, also encodes delay discounting^25^. In the mammalian brain, delay discounting and effort discounting are thought to be coded in separate areas, with delay discounting being attributed to the orbitofrontal cortex, and effort discounting to the ACC^8,10,29–31^. One possibility is that the smaller avian brain has evolved to carry out more generalised value computations in a single region, NCL. Another possibility is that we have yet to fully explore the possibility that NCL consists of sub-regions each serving the different aspects of processing reward-based information.

### Implications for CDL function

A handful of CDL cells were Effort Selective in that they fired during either LE or HE trials. However, unlike in NCL, as a population, cells in CDL showed no modulation in firing that reflected a preference for the LE or HE trials. Of course, we exercise some caution in this conclusion as we recorded from a smaller number of CDL cells than NCL cells. Although overall CDL cells seemed to exhibit no neural activity indicative of a preference for either the LE or HE trials, an interesting observation was noted in the number of inhibitory and excitatory cells in each period of the task. For example, 95% of CDL cells fired in an inhibitory manner during the 300 ms prior to the first effort peck, and 60% fired in an inhibitory manner during the Reward period. In contrast, during the Post-Effort period, 86% of cells fired in an excitatory manner. So while not firing differentially between HE and LE trials, a large proportion of CDL cells appeared to be inhibited during the Effort period, and then seem to ‘rebound’, firing in an excitatory manner during the Post-Effort period, before being again inhibited during the Reward period. Although neurons in CDL may not play a role in value coding, CDL cells could be involved in response-outcome coding, playing an important role in associating the effortful action of pecking to the beneficial reward outcome. If this is the case, CDL could still play an important role in learning and associating actions with outcomes.

It is interesting that despite the fact that CDL has some connectivity patterns similar to the ACC^23^, cells in CDL do not appear to directly modulate their firing rate in response to effort discounting in the same way that ACC does^8,9,29^. The current literature on the function of CDL is limited, and to our knowledge no other study has conducted electrophysiology recordings from CDL. CDL is part of the limbic/olfactory sub-module that makes up the cortico-hippocampal network of the avian brain^32^. The majority of studies that make mention of CDL are lesion studies, where CDL is used as a control area, and in most of these cases the lesion is imprecise and affects surrounding areas. As such, it is difficult to draw on any previous literature to speculate on why CDL exhibits the pattern of inhibitory and excitatory changes observed in the current study. We found that CDL does not seem to directly code value, but does seem to fire in a pattern that may be useful in associating action and reward. We therefore posit that CDL may be involved more in the updating of the mental model of an action-outcome sequence^33^ rather than representing the value of each outcome in the way that NCL does.

### Conclusions

We found the cells in the pigeon NCL encode value as a function of effort discounting. Our findings are consistent with a growing body of literature suggesting the NCL is an important area of the pigeon brain for encoding value^19–21,25^. Unlike the mammalian brain, the avian brain does not seem to process delay discounted value and effort discounted value in separate areas, but rather NCL acts as one more generalized value coding region. Despite some analogies between ACC and CDL in terms of connections patterns, while a handful of CDL cells were Effort Selective, we found no evidence that CDL cells encoded value. Nevertheless, it is still possible that CDL plays a role in associating responses and outcomes.

## Data Availability

Data will be made available upon request.
